# Temporal dynamic effects of meteorological factors and air quality on the physical health of the older adults in Shenzhen, China

**DOI:** 10.3389/fpubh.2024.1289253

**Published:** 2024-03-06

**Authors:** Shuai Jiang, Chuanliang Han, Yue Ma, Jiajia Ji, Guomin Chen, Yinsheng Guo

**Affiliations:** ^1^Shenzhen Center for Disease Control and Prevention, Shenzhen, Guangdong, China; ^2^Department of Electrical Engineering, The City University of Hong Kong, Hong Kong, Hong Kong SAR, China; ^3^Department of Healthcare-Associated Infection Management, National Clinical Research Center for Infectious Diseases, Third People’s Hospital of Shenzhen and The Second Hospital Affiliated to Southern University of Science and Technology, Shenzhen, Guangdong, China

**Keywords:** climate, pollution, physical examination, public health, Shenzhen

## Abstract

**Introduction:**

Meteorological and environmental factors can affect people’s lives and health, which is crucial among the older adults. However, it is currently unclear how they specifically affect the physical condition of older adults people.

**Methods:**

We collected and analyzed the basic physical examination indicators of 41 older adults people for two consecutive years (2021 and 2022), and correlated them with meteorological and environmental factors. Partial correlation was also conducted to exclude unrelated factors as well.

**Results:**

We found that among the physical examination indicators of the older adults for two consecutive years, five indicators (HB, WBC, HbAlc, CB, LDL-C) showed significant differences across the population, and they had significantly different dynamic correlation patterns with six meteorological (air pressure, temperature, humidity, precipitation, wind speed, and sunshine duration) and seven air quality factors (NO2, SO2, PM10, O3-1h, O3-8h, CO, PM2.5).

**Discussion:**

Our study has discovered for the first time the dynamic correlation between indicators in normal basic physical examinations and meteorological factors and air quality indicators, which will provide guidance for the future development of policies that care for the healthy life of the older adults.

## Introduction

Meteorological factors are one of the most important influencing factors on population health. Global warming, changes in meteorological conditions, extreme weather, and natural disasters pose a serious threat to human health, which can lead to cardiovascular and cerebrovascular disease, infectious diseases, injuries, and psychological/mental diseases (1–7). Changes in weather and environment are also thought to have a significant impact on the emergence and extinction of certain infectious diseases (8, 9). According to the WHO study on the health and economic impacts of climate change on populations between 2030 and 2050, it is estimated that the annual increase in deaths due to climate change worldwide is 250,000, with 38,000 older adults people (≥ 60 years old) dying from heat waves, 48,000 deaths from diarrhea and dysentery, and 60,000 deaths from malaria (10). Exploring the relevant factors of weather-sensitive diseases, forecasting and early warning of weather-sensitive diseases, and developing relevant intervention technologies to reduce the impact of weather factors on population health have become the hot spots of global studies (11).

The impact of the meteorological environment on human health has gradually become a focus of concern for the people (12–15), and it is also an urgent issue that the government and scientific research institutions need to solve. Some scholars collected the historical data of 15 European countries from 1990 to 2000 and made a comparative analysis of the unique temperature death effect relationship in each country. They found that high temperature had a more significant relationship with mortality caused by respiratory disease, while the mortality of the older adults was more sensitive to temperature changes (16). A multicenter study on temperature and mortality in 272 cities across 7 countries was published in 2015. Based on the collection of temperature data, the total number of deaths, meteorological conditions, and air pollutants, the quantitative relationship between temperature and mortality effects unique to each city was analyzed. It was found that there are differences in temperature death effect curves at the city scale (17). In addition, a study conducted in four cities in East Asia (Seoul, Beijing, Tokyo, and Taipei) also pointed out significant regional differences in the health effects of high temperatures, and regional differences should be considered in analyzing the impact of climate change (18). On the basis of ecological research, researchers have established prediction and early warning models for some weather-sensitive diseases by using a variety of mathematical analysis methods, such as generalized additive models and neural network analysis, to carry out spatiotemporal prediction of weather-sensitive diseases (19, 20), provide more accurate risk prediction information for decision-makers, and reduce the disease burden caused by the meteorological environment (21, 22). Since the 1990s, the United States and several European countries have established a heat wave early warning system based on air mass classification, which has effectively reduced the incidence rate and mortality of weather-sensitive diseases (23).

In Shenzhen, a coastal area with a changeable climate in China, the incidence rate of diseases closely related to meteorological factors is also on the rise year by year (24), such as respiratory diseases represented by upper respiratory tract infections and chronic obstructive pulmonary diseases, and circulatory diseases represented by hypertension and cardiovascular and cerebrovascular accidents (4, 6). However, it is still unclear how changes in meteorological and pollution factors affect the basic functional indicators of the human body. Whether it is meteorological factors or air quality, they often do not directly affect human health in the first place but require time to accumulate. However, currently, the impact of this delayed nature on physical condition is also unclear (Table 1).

**Table 1:** Statistical description of the sample.

Items	2021 (M ± Std)	2022 (M ± Std)	*P*
Temperature/ ^o^C	36.25 ± 0.17	36.21 ± 0.21	ns
Pulse rate/min /Hz	77.39 ± 12.88	77.29 ± 11.29	ns
Respiratory rate/min /Hz	18.05 ± 1.11	18.43 ± 0.87	ns
Height/ cm	160.07 ± 5.98	159.70 ± 6.64	ns
Weight/ kg	63.70 ± 11.02	64.50 ± 11.29	ns
Waist/ cm	86.18 ± 10.64	87.18 ± 10.59	ns
BMI/ kg/m^3^	25.01 ± 3.84	25.38 ± 3.69	ns
HB / g/L	149.41 ± 17.39	139.38 ± 11.06	<0.001
WBC/ 10^9^/L	5.58 ± 1.51	6.00 ± 1.28	0.0092
PLT/ 10^9^/L	503.74 ± 48.75	211.64 ± 49.87	ns
FBG/ mmol/L	5.41 ± 0.81	5.38 ± 0.78	ns
ALB/ mg/L	14.80 ± 14.48	18.34 ± 18.43	ns
Cr/ mmol/L	14,367 ± 8,592	16,070 ± 8,193	ns
HbAlc/ %	5.69 ± 0.38	5.79 ± 0.33	0.0026
CEA/ mg/L	1.93 ± 1.35	2.47 ± 1.39	ns
AFP/ mg/L	3.04 ± 1.23	2.84 ± 1.06	ns
SAT/ U/L	21.34 ± 8.33	21.66 ± 9.29	ns
SGOT/ U/L	22.89 ± 7.62	24.08 ± 6.42	ns
Tbil/ mmol/L	10.89 ± 5.29	11.47 ± 3.63	ns
CB/ mmol/L	3.64 ± 1.48	3.15 ± 1.03	0.0306
UB/ mmol/L	9.20 ± 4.48	9.25 ± 4.74	ns
SCR/ mmol/L	65.96 ± 12.23	67.14 ± 12.69	ns
BUN/ mmol/L	5.29 ± 1.23	5.66 ± 1.31	ns
UA/ mmol/L	339.13 ± 73.93	340.34 ± 80.88	ns
TC/ mmol/L	5.34 ± 1.01	5.39 ± 1.12	ns
TG/ mmol/L	1.87 ± 1.41	2.03 ± 1.40	ns
LDL-C/ mmol/L	2.81 ± 0.70	3.18 ± 0.88	0.0023
HDL-C/ mmol/L	1.47 ± 0.31	1.49 ± 0.35	ns

In this study, we aimed to link multiple meteorological variables (temperature, humidity, rainfall, pollutant, etc.) and indicators of human health in the older adults population. By comparing the physical examination indicators of the same group of older adults people before and after 2 years, we first found six of them to have significant differences. Further, we conducted a time-lag correlation analysis between the changes in these six indicators in the previous and second years and the weather and air pollution indicators from 1 to 90 days before the physical examination in order to obtain the temporal dynamic correlation between weather and pollution factors on health indicators.

## Methods

### Data collection - physical examination

Forty-one elder subjects (age = 69.93 ± 4.29, 18 M, 23F) participated in this study. All subjects are residents of Shenzhen and are Han nationals. In 2021, the physical examination of subjects is scheduled around April, and in 2022, it is scheduled from April to July at the local hospital in Nanshan District, Shenzhen.

The physical examination indicators included in the analysis are temperature (T, ^o^C), pulse rate (P, times/Min), respiratory rate (RR, times/min), height (cm), weight (kg), waist (cm), body mass index (BMI, kg/m^3^), hemoglobin (HB, g/L), white blood cells (WBCs, ×10^9^/L), platelets (PLTs, ×10^9^/L), fasting blood glucose (FBG, mmol/L), urinary microalbumin (ALB, mg/L), urinary creatinine (Cr, mmol/L), glycosylated hemoglobin (HbAlc, %), carcinoembryonic antigen (CEA, mg/L), alpha fetoprotein (AFP, mg/L), serum alanine transaminase (SAT, U/L), serum glutamic oxaloacetic transaminase enzyme (SGOT, U/L), total bilirubin (TBil, mmol/L), conjugated bilirubin (CB, mmol/L), unconjugated bilirubin (UB, mmol/L), serum creatinine (SCR, mmol/L), blood urea nitrogen (BUN, mmol/L), blood uric acid (UA, mmol/L), total cholesterol (TC, mmol/L), triglyceride (TC, mmol/L), serum low-density lipoprotein cholesterol (LDL-C, mmol/L), and serum high-density lipoprotein cholesterol (HDL-C, mmol/L).

### Data collection - meteorological factors

From the meteorological department of Shenzhen, integrating and collecting relevant data from existing monitoring platforms and on-site investigation data, we obtained daily meteorological factor data for 2021 and 2022. The meteorological factors include average air pressure (hpa), average temperature (°C), average humidity (%), precipitation (mm), average wind speed (m/s), and sunshine duration (hours/day).

### Data collection – air quality

From the meteorological department of Shenzhen, we obtained daily air quality data for 2021 and 2022. The meteorological factors include NO2 (ug/m^3^), SO2 (ug/m^3^), PM10 (ug/m^3^), O3-1h (ug/m^3^), O3-8h (ug/m^3^), CO (mg/m^3^), and PM2.5 (ug/m^3^).

### Ethics statement

The study was based on a long-term stable follow-up community queue, and approved by the Ethics Committee of Shenzhen Center for Disease Control and Prevention.

### Correlation analysis

We conducted Pearson’s correlation between the change of meteorological factors and the change of physical examination indicators in 1 to 90 days’ delay, which aimed to explore the number of days before the physical examination and whether the weather would affect the indicators on the day of the examination. We used the average of environmental indicators corresponding to the delay of 1 to 90 days before the physical examination date; for example, the correlation when the delay time is 10 means the ratio of changes in physical examination indicators over 2 years (log ratio) and the ratio of changes in the average of environmental indicators within 1 to 10 days before the physical examination date (log ratio). This idea was also applied to calculate the relationship between changes in air quality and changes in physical examination indicators. The change in index was calculated as the logarithm of the ratio of value in 2022 and that in 2021. In a few cases, a particular indicator may be significantly correlated with multiple factors. In these cases, we further use the partial correlation analysis method to exclude irrelevant factors.

### Statistical analysis

A pair-wise t-test was used to compare the difference between 28 indicators from the physical examination in 2021 and 2022.

## Results

**Figure 1:**
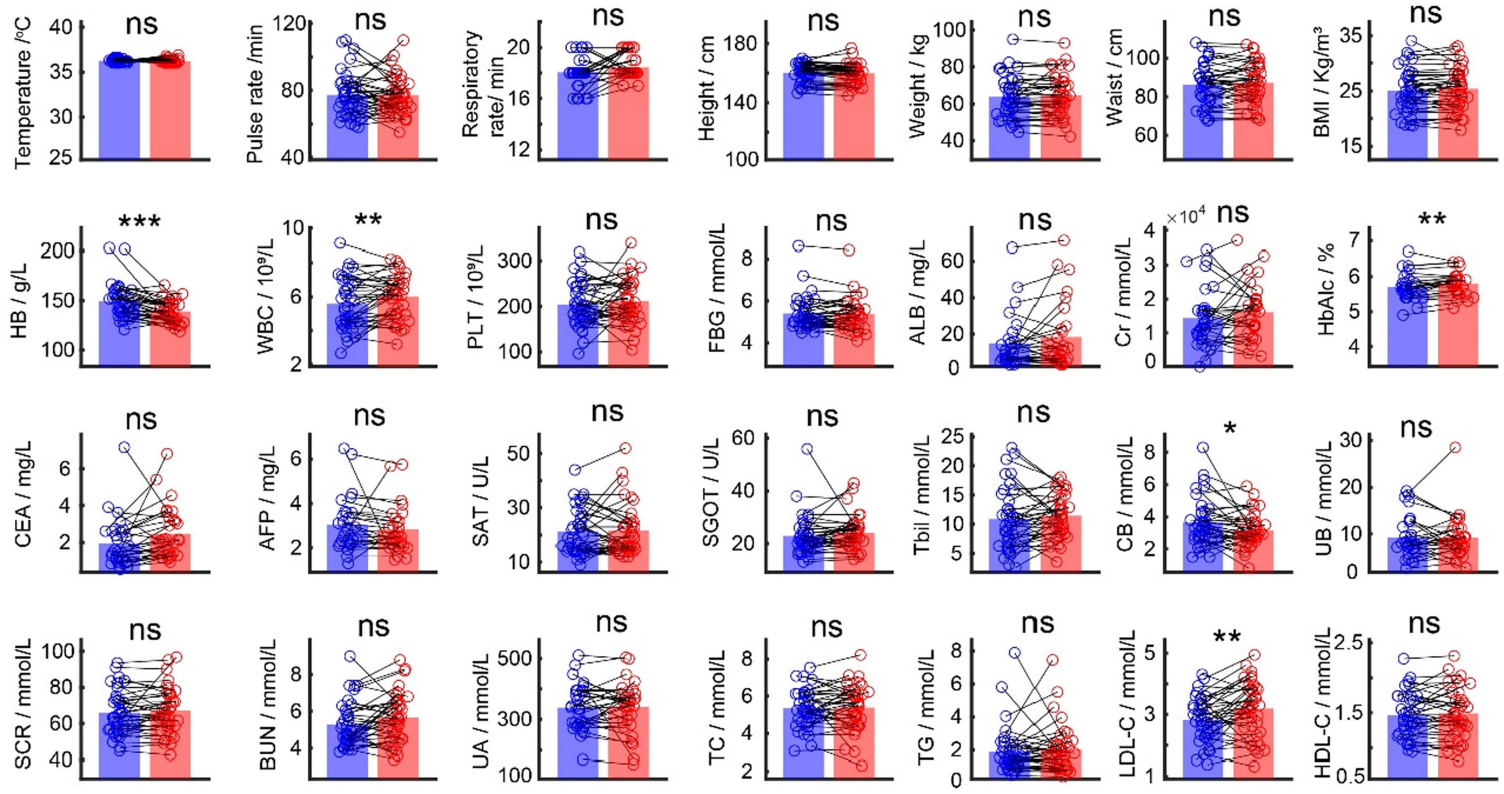
Comparison between the physical examination indicators in 2021 and 2022. Blue bars are average values for each indicator in 2021, and red bars are average values for each indicator in 2022. Black dots are individual values each indicator.

**Figure 2:**
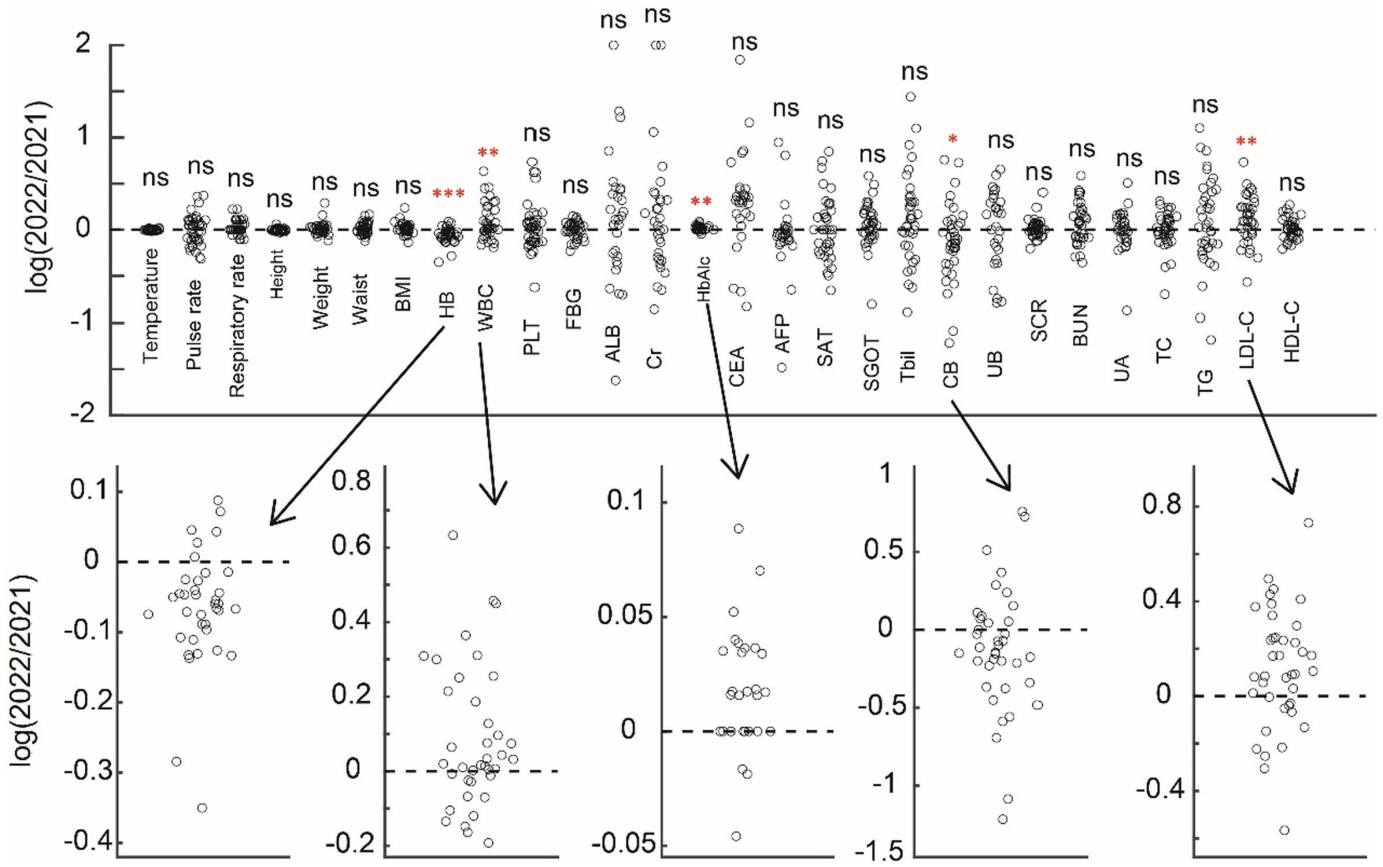
Comparison between the physical examination indicators in 2021 and 2022 in the ratio form of the demonstration. Above subfigure shows the change of indicators, and five significant indicators are shown below.

We first compared the basic 28 physical examination indicators of 41 older adults people for 2 consecutive years (Figure 1) and found that five of them had significant changes (HB, WBC, HbAlc, CB, and LDL-C). Among them, HB (*p* < 0.001) and CB (*p* = 0.031) were significantly reduced, while WBC (*p* = 0.009), HbAlc (*p* = 0.003), and LDL-C (*p* = 0.0023) were significantly increased (Figure 2).

**Figure 3:**
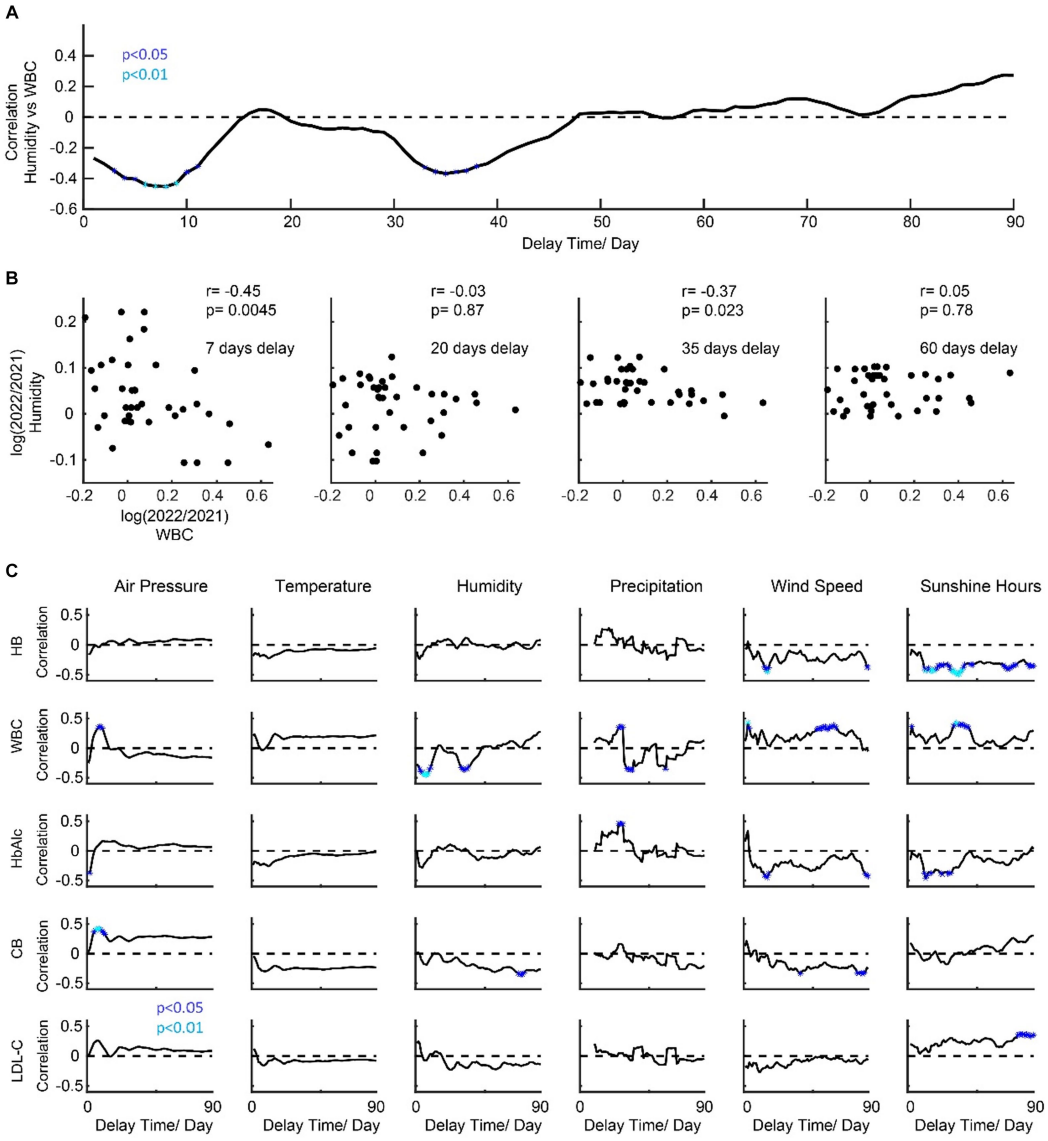
Temporal dynamics of the correlation patterns for physical examination indicators and meteorological factors. **(A)** shows a demo for dynamic correlations (humidity vs. WBC). **(B)** shows a scatter plot for four timing points. **(C)** shows the temporal dynamics of the correlation patterns for physical examination indicators and meteorological factors.

In order to further explore the potential mechanism of the change of five main indicators, we conducted a correlation analysis with meteorological factors (Figure 3) and different delay times for the change of five main indicators. A demo is shown in Figure 3A, which illustrates the temporal dynamics of the correlation between humidity and WBC over 90 days. In this example, the humidity index around days 8 and 35 before the physical examination would be negatively correlated with the WBC indicator, while for the other days, the correlation is not significant (Figure 3B). For all five indicators (HB, WBC, HbAlc, CB, and LDL-C), we found that they have distinct patterns for the correlation’s curves to six meteorological factors (air pressure, temperature, humidity, precipitation, wind speed, and sunshine duration) (Figure 3C). From the result, HB is negatively correlated with wind speed and sunshine. WBC is negatively correlated with the humidity but positively correlated with the wind speed and sunshine hours, and it is notable that the polarity of correlation changes in precipitation. HbAlc is positively correlated with the precipitation but negatively correlated with the wind speed and sunshine hours. CB is negatively correlated with wind speed and humidity. LDL-C is positively correlated with sunshine hours.

**Figure 4:**
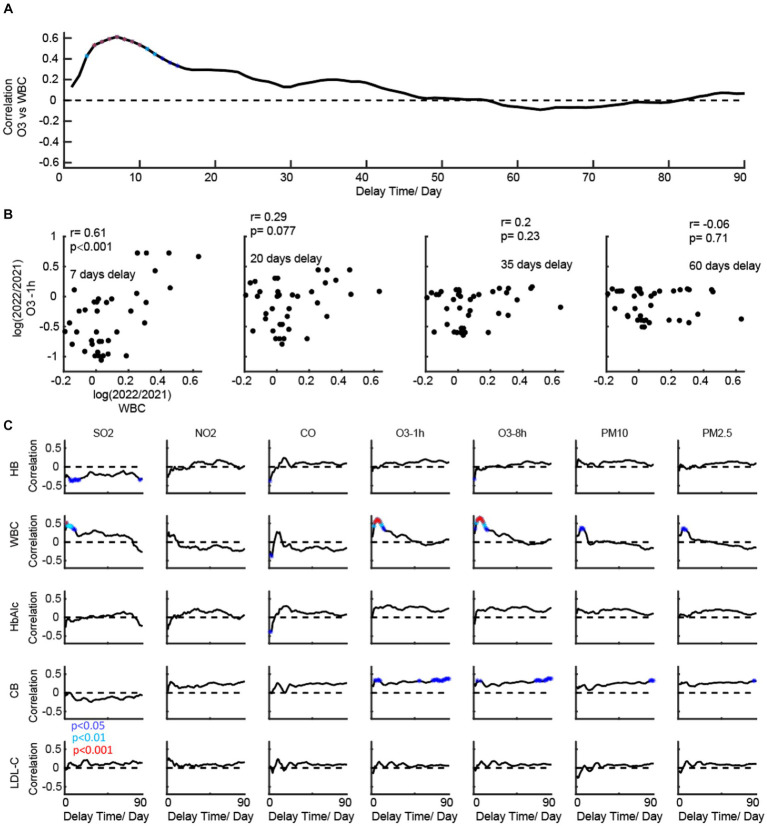
Temporal dynamics of the correlation patterns for physical examination indicators and air quality. **(A)** shows a demo for dynamic correlations (O3 vs. WBC). **(B)** shows a scatter plot for four timing points. **(C)** shows the temporal dynamics of the correlation patterns for physical examination indicators and meteorological factors.

We then conducted a correlation analysis of air quality (Figure 4) with different delay times for the change of five main indicators. The ideas of the demo in Figures 4A,B are similar to those in Figure 3. We found that they have distinct correlation patterns for the correlation’s curves to seven air quality indices (Figure 4C). HB is negatively correlated with SO2. WBC is positively correlated with SO2, O3, PM2.5, and PM10. CB is positively correlated with O3. HbAlc and LDL-C did not show any significant correlation with the air quality indices.

**Figure 5:**
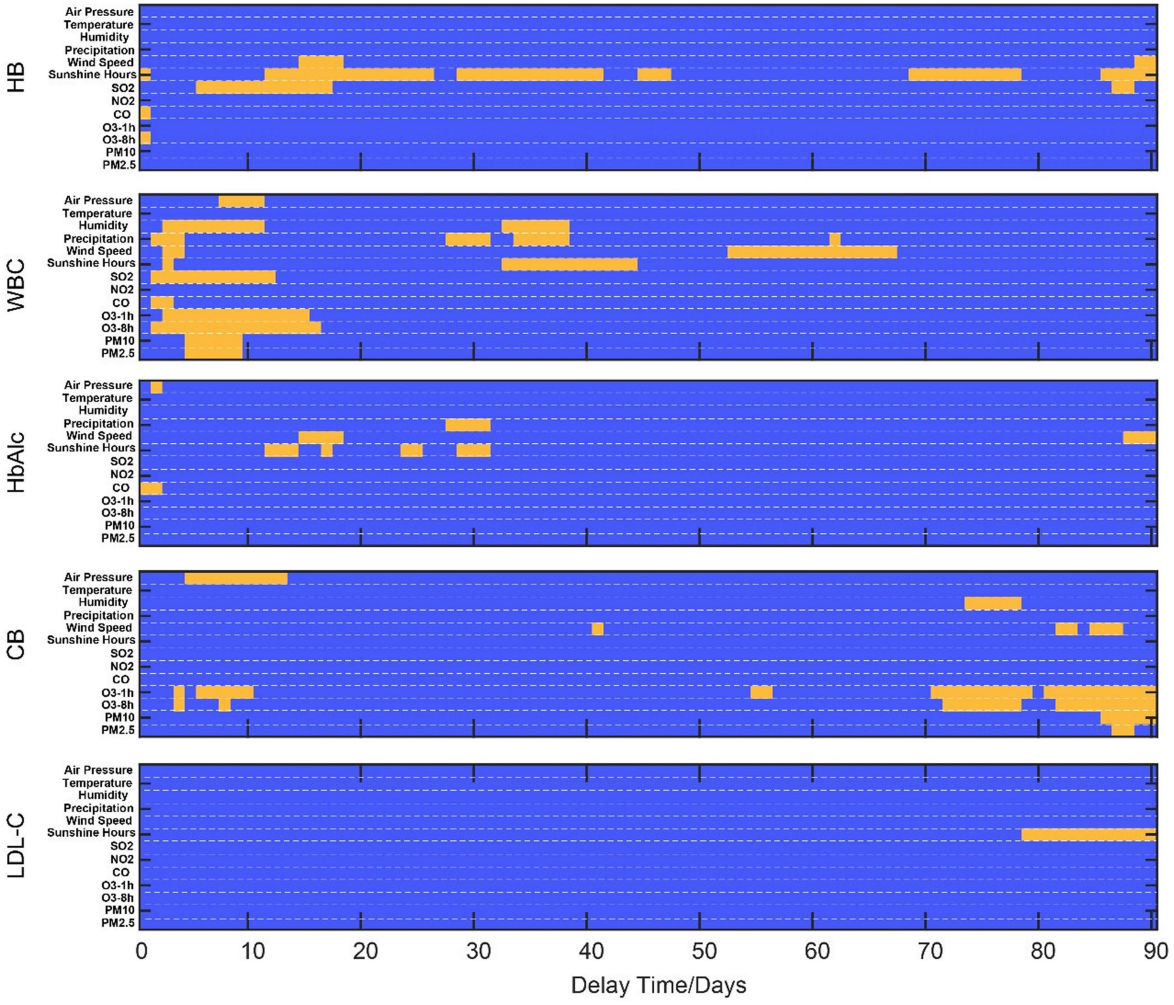
Demonstration of significant correlation time ranges for physical examination indicators and natural indices. Blue regions show an insignificant correlation between physical examination indicators and natural indices. Yellow regions show a significant correlation between physical examination indicators and natural indices.

It is also noteworthy that, in some cases, a specific indicator may be significantly correlated with multiple factors (Figure 5). In such cases, we further employ the partial correlation analysis method to eliminate irrelevant factors. The changes in sunlight duration, CO, and O3 have an immediate impact on HB. After using partial correlation analysis to identify the factors associated with this immediate impact, we found that only the change in sunlight duration was significantly correlated with the change in HB (*p* = 0.0225), after controlling for the other two variables. Meanwhile, the changes in wind speed, sunlight, and SO2 have a medium (*p* < 0.001) and long-term impact (*p* < 0.0318) on HB. Through partial correlation analysis, we discovered that only the change in sunlight duration was significantly correlated with the change in HB. In summary, the main factor affecting the change in HB is the change in sunlight duration. For WBC, pressure, humidity, precipitation, wind speed, sunshine, SO2, CO, and O3 all have short-term immediate effects on it. Through partial correlation analysis, we found that only SO2 has a significant short-term effect (*p* = 0.049). Humidity, precipitation, and sunshine also have a medium-term impact on it. Through partial correlation analysis, we found that precipitation is mainly a factor (*p* < 0.001), and wind speed has a long-term impact (*p* = 0.038).

Precipitation, wind speed, and sunshine have a medium-term impact on HbAlc. Through partial correlation analysis, we found that only changes in precipitation have a significantly weak correlation effect on its change (*p* = 0.0289) after excluding other indicators. For CB, although there are some significant correlations (air pressure, wind speed, and O3, PM) through relevant analysis, these correlations become non-significant after partial correlation analysis. For LDL-C, only sunlight exposure duration has a long-term positive impact, which remains significant even after correction for confounding.

## Discussion

Our study, taking Shenzhen city as an example for the first time, explores the dynamic correlation between physical examination indicators of the older adults and meteorological factors and air quality. This study has guiding significance for how the weather environment affects older adults health indicators.

### Comparison with previous studies

There have been previous studies on the relationship between meteorological factors and the occurrence of different diseases (4, 6, 12, 24–30). A large number of time series studies and cross-case studies based on ecological research design have basically elucidated the exposure–response relationship between meteorological conditions and sensitive diseases in developed countries in Europe and America (31–33). Previous epidemiological studies have found that significant changes in meteorological factors such as temperature, humidity, and precipitation can alter the exposure level of the population to certain meteorological factors, thereby increasing the risk of illness, injury, and death (12, 34–37); The change in meteorological environment can create suitable conditions for the parasitism, reproduction, and transmission of mosquitoes and other vectors and parasitic pathogens, and change the density and seasonal distribution of parasites and vectors, thus affecting the epidemic process of insect vectors and parasitic infectious diseases, expanding the epidemic degree and scope, and increasing the harm to the population (9, 38–41).

Research in China also shows that meteorological factors can also affect the incidence rate of infectious diseases, such as bacillary dysentery (42) and hand-foot mouth (43, 44). In addition, extreme weather events can also induce some chronic non-infectious diseases (such as cardio-cerebral vascular disease, respiratory disease, genitourinary system disease, and metabolic system disease), injuries (such as traffic accidents, drowning, and suicide), psychological/mental diseases (such as depression, schizophrenia, and emotional and behavioral abnormalities), and adverse pregnancy outcomes (28, 33, 45–47). China has also conducted some research on weather-sensitive diseases, mainly based on changes in meteorological factors and long-term changes in the number of emergency outpatient services, inpatients, and deaths. The time series method and case-crossing method have been applied to study the correlation between meteorological environment and population health, and epidemiological evidence has been obtained on the meteorological factors and population health effects of some diseases. It is concluded that the change in meteorological conditions has a significant impact on common cardio cerebral vascular disease (such as hypertension, stroke, coronary heart disease, and myocardial infarction), respiratory disease (colds, chronic obstructive pulmonary disease, asthma, and bronchitis), as well as rheumatism, diabetes, peptic ulcer disease, and other diseases (48). Some studies also point out that meteorological factors and air pollutants have synergistic effects on the mortality of various diseases and the incidence rate of certain diseases (such as acute coronary syndrome) (13), which may affect the emission, transfer, diffusion, and chemical transformation of pollutants due to changes in meteorological conditions or residents’ exposure patterns to pollutants in different seasons. Our findings reveal that sunlight exposure has different effects on HB and LDL-C, which may be related to some biochemical reactions involved in sunlight exposure. The relationship between WBC and natural factors is complex, as it is an essential component of the immune barrier in humans and is also influenced by various different factors, some of which may indeed have an impact on human immunity.

### Limitations and future study

We admitted that the relationship between various physiological indicators in humans and environmental factors is complex. An epidemiological study with more time points is needed for a proper definition of correlation. Physiology is not exclusively determining the health status, and pollution/weather may also interact via the psyche. More detailed research will be needed in the future to further answer this question.

## Data availability statement

The original contributions presented in the study are included in the article/supplementary material, further inquiries can be directed to the corresponding author/s.

## Ethics statement

The studies involving humans were approved by the Ethics Committee of Shenzhen Center for Disease Control and Prevention. The studies were conducted in accordance with the local legislation and institutional requirements. The participants provided their written informed consent to participate in this study.

## Author contributions

SJ: Conceptualization, Data curation, Investigation, Methodology, Writing – original draft, Writing – review & editing. CH: Conceptualization, Data curation, Formal analysis, Investigation, Methodology, Validation, Visualization, Writing – original draft, Writing – review & editing. YM: Data curation, Investigation, Writing – review & editing. JJ: Writing – review & editing. CG: Writing – review & editing. YG: Conceptualization, Formal analysis, Funding acquisition, Investigation, Methodology, Project administration, Validation, Writing – review & editing.
